# Hemispatial Neglect Shows That “Before” Is “Left”

**DOI:** 10.1155/2016/2716036

**Published:** 2016-05-29

**Authors:** Mario Bonato, Arnaud Saj, Patrik Vuilleumier

**Affiliations:** ^1^Department of Experimental Psychology, Ghent University, 9000 Ghent, Belgium; ^2^Department of Clinical Neurosciences, Neuropsychological Unit, University Hospital of Geneva, 1205 Geneva, Switzerland; ^3^Department of Neurosciences, Laboratory of Neurology and Imaging Cognition, University of Geneva, 1211 Geneva, Switzerland

## Abstract

Recent research has led to the hypothesis that events which unfold in time might be spatially represented in a left-to-right fashion, resembling writing direction. Here we studied fourteen right-hemisphere damaged patients, with or without neglect, a disorder of spatial awareness affecting contralesional (here left) space processing and representation. We reasoned that if the processing of time-ordered events is spatial in nature, it should be impaired in the presence of neglect and spared in its absence. Patients categorized events of a story as occurring before or after a central event, which acted as a temporal reference. An asymmetric distance effect emerged in neglect patients, with slower responses to events that took place before the temporal reference. The event occurring immediately before the reference elicited particularly slow responses, closely mirroring the pattern found in neglect patients performing numerical comparison tasks. Moreover, the first item elicited significantly slower responses than the last one, suggesting a preference for a left-to-right scanning/representation of events in time. Patients without neglect showed a regular and symmetric distance effect. These findings further suggest that the representation of events order is spatial in nature and provide compelling evidence that ordinality is similarly represented within temporal and numerical domains.

## 1. Introduction

Mounting evidence suggests that time is represented in spatial terms [1]. Tversky and collaborators [2] were among the first to show that the spontaneous spatial positioning of temporally ordered events follows the direction of reading/writing. Also overlearned sequences of items, either temporally characterized (days of the week) or nontemporally characterized (letters of the alphabet), can interact with the spatial position of response keys [3, 4]. This interaction has often been described, within a given sequence, in terms of faster left-sided responses for earlier than for late items and, viceversa, in terms of faster right-sided responses for late than for earlier items. An association between ordinal value and lateralized responses can also be found for items whose order is newly and arbitrarily learned [5–7].

The method and the interpretation of these studies (all with healthy participants) have been however strongly criticized, among others, by Proctor and Cho [8]. According to their influential “polarity correspondence” theory, the space-order association in categorization tasks would be a byproduct of a structural, and not conceptual, overlap between the code used to categorize the item and the code used for providing responses. By testing the performance of patients who are affected by spatial processing disorders, it is possible to directly determine whether they show symmetric performance (as predicted by the polarity correspondence) or whether some distortions occur, therefore supporting the view that the representation of ordered items is truly spatial in nature. To this aim, the present study compared the performance of right hemisphere damaged (RHD) patients with and without a deficit in processing the contralesional space called hemispatial neglect or unilateral spatial neglect (henceforth: neglect). In neglect, the items which are present in the ipsilesional hemispace are not as efficiently processed (and sometimes completely ignored) as those presented in the ipsilesional hemispace [9–13]. Neglect patients offer the unique possibility to study the nature of order representation by contrasting a pathological performance for the contralesional hemispace with a less impaired (or close to normal) performance for the ipsilesional hemispace.

The idea of studying whether specific deficits for time processing are present in neglect is not new. Neglect patients have been already described to suffer from deficits in the processing of time-related aspects (for review, see [14]). Most of the previous reports, however, focused on slowed dynamics and on a number of collateral aspects that can be affected by several, heterogeneous and not necessarily spatial, factors. Only recently, some studies directly assessed whether the deficits neglect patients show in time processing support a functional analogy between impairments in the spatial and time domains [1, 15, 16]. One line of research in RHD patients focused on experienced time by presenting short duration stimuli (typically below two seconds) to be either reproduced/bisected or categorized with respect to a reference. For instance, in a temporal bisection task, neglect patients showed a severe underestimation for stimuli duration [17]. This study suggests a close analogy between the deficits they show in physical space and those they manifest in processing time durations [1]. The same group of neglect patients also showed a reduction of their underestimation following prismatic adaptation (PA) which generated a leftward compensatory aftereffect. This means that spatial attention, which is deficient in patients and modulated by PA, is probably the medium for spatially representing time. Moreover, in the same study, both RHD patients without neglect and healthy controls showed temporal underestimation following the same PA procedure, further suggesting a causal involvement of spatial attention when elapsing time has to be processed and quantified [18]. These findings suggest that the way time is processed is truly spatial in nature, leading to the proposal that time might be represented along a mental line [1]. The transient modulation induced by PA in time processing for RHD patients with neglect proves that their bias in time processing is not due to unspecific cognitive impairments [19] and points to their deficit in unilateral orienting of visuospatial attention as a key determinant for their performance.

Two studies have shown that neglect patients present selective deficits also when brief time durations have to be categorized with respect to a standard. In a first one [20], RHD patients with neglect performed significantly worse than RHD patients without neglect and healthy controls, irrespective of the duration of the standard tone and without any interval-specific deficit. In a second study [21], patients with more severe neglect showed a disrupted time-space association for short durations only, as if these were ignored because of a left-to-right ordering. In contrast, [22] showed, within a group of right brain damaged patients, a neglect-specific difficulty when the quantity of nonsymbolic items had to be compared to a reference quantity but not when the comparison had to be done with respect to a reference duration. All in all, previous studies on neglect and time durations have reported rather conflicting results [22].

Still other studies reported that, even in the absence of neglect on paper-and-pencil tasks, RHD patients may be particularly slow when detecting contralesional targets after having been exposed to future tenses, which are supposed to orient spatial attention rightward [23]. The presence of a spatiotemporal bias at a sensitive computer-based testing in the absence of neglect at (not so sensitive) paper-and-pencil tests is not surprising [24–26]. At the same time, however, it should be noted that, in the absence of neglect at paper-and-pencil tests, right hemisphere damage does not necessarily result in deficits when processing different time durations [15].

While converging evidence suggests a role for spatial attention in processing time durations, the possibility that neglect might also affect the processing and representation of more conceptual aspects of time is almost totally unknown. One recent study [16] showed that left hemispatial neglect results in distortions of the sequential ordering of time-related events/features. In that investigation [16], the crucial time aspect was not related to duration but to conceptual/order-related aspects. Right brain damaged patients memorized a list of events/features they were told occurred in the past or would occur in the future. Patients with neglect mislabeled items belonging to the past as being “future” items both in recall and in recognitions tasks significantly more than participants without neglect did. This spatial distortion closely mirrors spatial mislocalizations in the visual space whereby contralesional targets, under demanding tasks, are reported to appear in the ipsilesional space [27].

In the context of our current approach to these issues, it is important to mention that, also in the numerical domain, neglect patients often present some distortions resembling their deficit in contralesional space processing. They show, in magnitude comparison tasks, pathological slowing for the number immediately smaller than a reference (e.g., 4 if the reference is five; or 6 if the reference is 7); see [35]. This slowing only appears in magnitude comparison tasks and does not emerge in parity tasks [36, 37]. A principal component analysis [37] revealed that this asymmetric distance effect cooccurred with rightward shift in line bisection within one component and with numerical interval bisection/parity judgment within a different one.

This slowing is reduced by a cloud of dots showing leftward motion [38], extending, according to the authors, the effects of optokinetic stimulation (OKS) to representational numerical neglect (see also [39, 40]). All these findings converge in showing that attentional biases, due either to neglect or to an attentional manipulation, may affect representations of number and time.

In the present study, we designed a new task requiring a binary, lateralized left/right response to categorize items belonging to a sequence of temporally ordered newly learned events. This choice allowed us to avoid any confounding due to potentially interfering long term associations, while lowering processing load. The task we adopted was inspired by the methodology described in [35] to study numerical representations. We not only aim to demonstrate a causal role of spatial attention in serial order processing but are also willing to prove that the consequences of damage to the neural and cognitive circuits devoted to spatial processing can extend to the domain of online-ordinal/conceptual time.

## 2. Materials and Methods

### 2.1. Sample

The only criterion for inclusion in the study was the presence of a single stroke within the right hemisphere. Exclusion criteria were dementia, substance abuse, and presence of other neurological disorders. We tested in total fourteen right hemisphere damaged patients, all right-handed (Table 1). Two patients were admitted to the Rehabilitation Center of Conselve (Padova, Italy) and were Italian speaking. The remaining twelve patients were tested in the University Hospital of Geneva (Switzerland) and were French speaking.

### 2.2. Neuropsychological Testing

Patients from Italy (*n* = 2) were tested with a battery for neglect (Conventional Part, Behavioural Inattention Test, BIT) [34] including three cancellation tasks (Lines, Letters, and Stars) and four copying and three drawing from memory tasks, plus a line bisection subtest. The two patients tested were both considered as having neglect because of a total battery score below the cut-off (129).

Patients from Switzerland (*n* = 14) were considered to present neglect when their performance was pathological in at least two of the three following tests: line bisection [28] (cut-off score: rightward deviation > 11%), scene copying [31] (cut-off: 1 out of 4), and bell cancellation [30] (cut-off for left omissions: 2 out of 15).

According to these criteria, eight patients entered the neglect group (N+: mean age 61.1 ± 7.8 years) and six patients entered the nonneglect group (N−: 57.4 ± 14.8 years). Average time from stroke was 81.7 ± 53 days for N+ and 51.5 ± 39 days for N− patients.

The study protocol was approved by the Ethical Committee of the Department of General Psychology, University of Padova, and the Central Ethics Committee of the University Hospital of Geneva. Patients signed informed consent to take part in the study.

#### 2.2.1. Lesion Neuroanatomy

**Figure 1:**
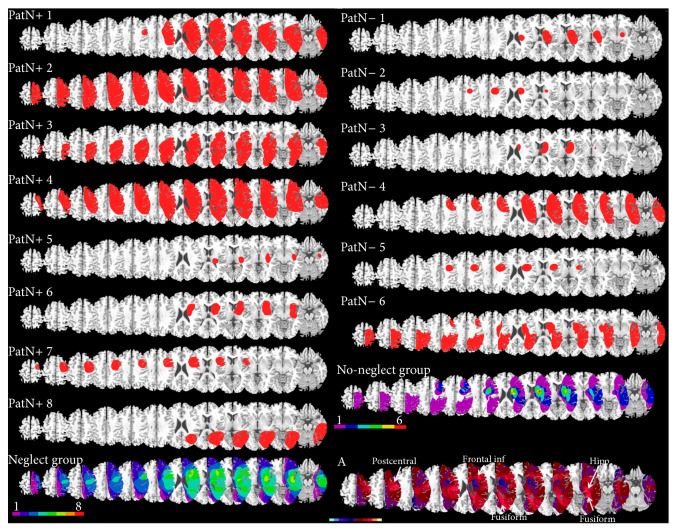
Individual lesion mapping (in red) and group overlap for neglect patients (N+, left) and no-neglect patients (N−, right). Lesion reconstruction was performed on axial slices of a normalized magnetic resonance imaging brain template. In the group overlap colors code for the number of patients with damage to a given area (from 1 = violet to 6 (N−) or 8 (N+) = red). (A) Median split subtraction analysis, comparing the lesion in patients with spatial neglect versus without. Each color in the scale bar codes for a 16.67% frequency of lesion in one or the other group, except for the central purple color that represents −16.67 to +16.67%.

For each patient, brain lesions were confirmed by brain scans (MRI or CT) and reconstructed on axial slices using MRIcro [41], according to previously described methods [42–44]. Lesioned areas were transformed to a three-dimensional region of interest (ROI) corresponding to the lesion volume and normalized to a standard brain template using MRIcro and SPM. The normalized lesion ROIs were then superimposed on a T1 MRI in order to determine the overlap of lesions across patients and define brain areas where damage was most commonly observed (Figure 1).

The mean lesion volume (the total number of lesioned voxels on the MRIcro brain template) did not significantly differ across the two groups (*p* = 0.15).

A direct contrast between these two groups using a voxel-wise subtraction analysis (Figure 1) indicates that damage to lateral frontal and temporal cortex was more commonly found in patients with spatial neglect (purple-yellow), whereas lesions in patients without neglect were centered on the pulvinar and the deep parietal white matter and medial temporal lobe (dark blue-turquoise).

### 2.3. Experimental Task

Patients were asked to pay attention to a story which was read aloud by the experimenter until the patient stated he/she succeeded in memorizing it.

The story (in Italian/French) was about a guy named “Giorgio.” Its translation is as follows: “Giorgio was smiling while riding his bike. The sun was shining in the sky. Tired and sweated, Giorgio jumped down from his bike and started to push it while holding a bottle. He arrived over the top of the mountain and looked to the city below him. [This was later identified as the reference event]. Then Giorgio started a fast downhill among clouds of dust. Giorgio failed to control his bike and fell inside a bush. His fall ended over the hood of a car that was passing by.”

Then the computer-based experiment was administered. Each trial started with a fixation cross (500 ms) followed by a blank screen (500 ms) and finally by an image representing one of the six events mentioned in the story (until response). Patients were informed that the reference event was the arrival of Giorgio at the top of the mountain and that they had to determine whether the image referred to an event that occurred before (images −3, −2, and −1) or after (images 1, 2, and 3) this reference event.

To overcome potential effects of neglect on visual perception, the image (92 mm wide and 45 mm high) was presented in the center of the screen. Since eye to screen distance was about 60 cm, image borders were relatively central (<5° of left/right eccentricity). The choice to present visual material was preferred after careful consideration of alternative possibilities: presenting target stimuli in auditory format would have strongly biased participants towards the use of verbal strategies for memorization. No image had critical information located on its extremities (neither left-sided nor right-sided).

**Figure 2:**
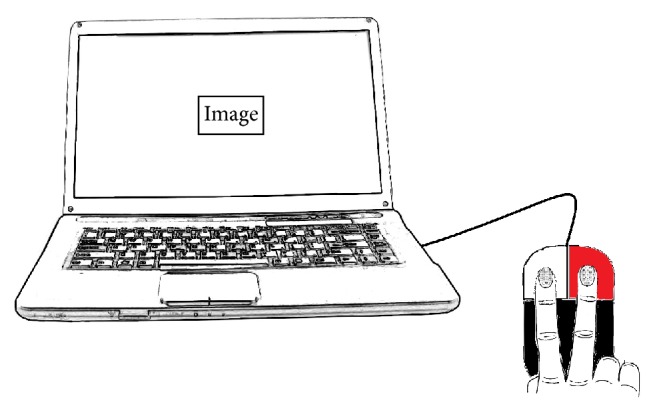
Experimental setup. Patients had to determine (index versus middle finger of the right hand) whether the presented image referred to an event occurring before or after the “central” event of the story, acting as temporal reference (adapted from http://jn.physiology.org/content/78/1/117 and from a drawing by Nita Jatar Kulkarni).

To overcome left-side hemiplegia, responses were implemented by using a mouse (Figure 2) with the right hand (index finger/left and middle finger/right; see [35, 45]). Each response key was arbitrarily assigned one colour (left white versus right red). This was done to avoid the possibility that spatially characterized words in the instructions about key presses would have led to arbitrary spatial associations. An auditory feedback (low tone) was given in case of wrong response. There were 12 repetitions for each condition and for each mapping, for a total of 144 trials for each patient. The monitor was positioned at a viewing distance of about 60 cm.

There was a practice run of 12 trials which was repeated if needed. For performing the second experimental block, patients were asked to switch the response keys; a second practice was again performed before performing the experimental trials with the new mapping. The initial mapping (“before” response assigned to the left key and “after” assigned to the right key) or vice versa was counterbalanced between participants.

## 3. Results

### 3.1. Reaction Times

**Figure 3:**
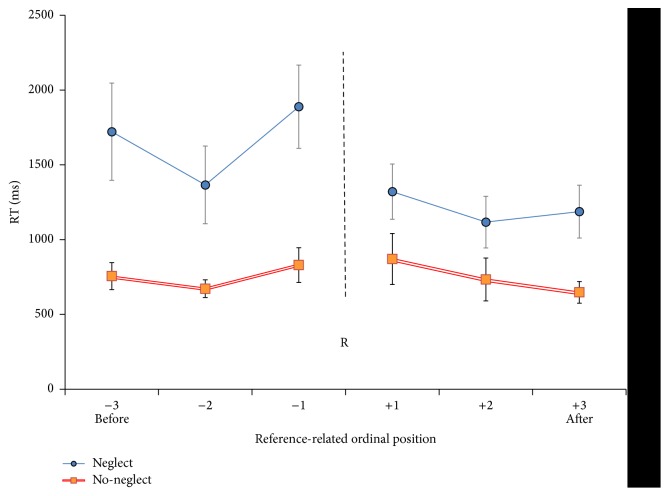
Distance effect. RTs as a function of the presented image are shown. In the N+ group (upper line, in blue) the Distance effect (slower RTs when closer to the reference) is asymmetric for items before versus after the reference whereas in N− (lower line, in red/orange) it is symmetric. The “position” of the reference event is represented by the letter “R.” Bars index SEM.

Before analysing RTs, we checked for overall accuracy (analysis on error rate is reported later). One neglect patient (N+ 3) failed to achieve with instructions and had to be excluded from the study. Neglect patient N+ 2 presented an error rate in the DP mapping too high (68%) to consider his reaction times reliable. We therefore analysed his median RTs from the PD mapping only, where his performance was better (less than 30% of errors). Reaction times for correct responses in 13 patients entered an ANOVA with Time (before versus after the reference) and Distance (1 versus 2 versus 3) as within-subject factors, plus Group (N+ neglect present versus N− neglect absent) as between-subjects factor (Figure 3). For each patient, condition, and mapping, medians were calculated and then averaged across mappings.

The main effects of Time, *F*(1,11) = 5.9, *p* < 0.05,  and  *η* ^2^ = .35, and Distance, *F*(2,22) = 7.8, *p* < 0.01, and *η* ^2^ = .42, were significant. The Group effect was also significant; neglect patients were overall slower: *F*(1,11) = 7.7, *p* < 0.05, and *η* ^2^ = .41 (N+: 1433 ms versus N−: 751 ms).

These effects were qualified by the crucial Time × Group interaction, indexing slowing for items before the reference which occurred in neglect patients only: *F*(1,11) = 6.6, *p* < 0.05, and *η* ^2^ = .38 (N+ before = 1659 ms; N+ after = 1208 ms; N− before: 752 ms; N− after: 751 ms). The Distance × Group interaction failed to reach significance: *F*(2,22) = 1.8, *p* = 0.18, ns.

To better understand the pattern determining the interaction, two separate ANOVAs, one for N− and one for N+, were performed with the same within-subjects factors as above.

In N− group, only the main effect of Distance was significant: *F*(2,10) = 4.6, *p* < 0.05, and *η* ^2^ = .5. It reflected slower and symmetric (no interaction with “Time”) responses for items closer to the reference (*d*1 = 850 ms; *d*2 = 703 ms; *d*3 = 702 ms).

In N+ group, the main effect of Distance was also significant: *F*(2,12) = 5.58, *p* < 0.05, and *η* ^2^ = .48. In addition, as predicted, a main effect of Time emerged, *F*(1,6) = 7.9, *p* < 0.05, and *η* ^2^ = .57, indicating an asymmetry for items presented before versus after the reference. The Time × Distance interaction was not significant: *F*(2,12) = 1.11, *p* = 0.36, and *η* ^2^ = .16. Inspection of data revealed that the main effect of Time was due to slower responses to events occurring before the reference (average before = 1659 ms; average after = 1208 ms). The slowing was seemingly irregular across the different distances [*d*] (before items: *d*1 1889 ms; *d*2 1366 ms; *d*3 1721 ms).

To investigate our specific hypothesis on the presence of a selective asymmetry in neglect involving items before versus after the reference, we performed, separately for N+ and N− patients, one *t*-test for each of the three temporal distances. A significant slowing emerged, in the N+ group only, for the item immediately before the reference versus the item immediately after [*t*(6) = 4.2, *p* < 0.01], as well as for the first versus the last item [*t*(6) = 2.39, *p* < 0.05 (one-tailed)]. Differences were particularly large (about half second) for both distances 1 (before 1889 ms; after: 1321 ms) and 3 (before 1721 ms; after: 1187 ms).

### 3.2. Different Mappings

Finally, an ANOVA with response Side as additional factor was performed. The main effects of Time and Distance and the Time × Group interaction were still present. Moreover a significant Time × Side interaction (SNARC-like [46]) emerged: *F*(1,10) = 10.96, *p* < 0.001, and *η* ^2^ = .52. Average RTs for events occurring “before” the reference were 950 ms for left-sided and 1255 ms for right-sided responses; for events occurring “after” the reference, the average RTs were 796 ms for right-sided responses and 1090 ms for left-sided ones. The three-way interaction Time × Side × Group was not significant: *F* = 1.44 and *p* = 0.26, suggesting that the spatial coding of events order similarly interacted with the response side in both neglect and nonneglect patients. Overall, no significant effect of response side emerged: *F*(1,10) = .006 and *p* = 0.94 (left 1020 ms versus right 1025 ms). When considering the two groups separately, the two-way Time × Side interaction turned out to be significant in neglect only (N+: *F*(1, 5) = 7.23, *p* < 0.05, *η* ^2^ = .59; N−: *F*(1, 5) = 3.75, *p* = 0.11, *η* ^2^ = .43). A pattern suggesting the presence of a Time (before/after) × Side (left-sided versus right-sided response) interaction was however clearly present in both groups (N+: left before 1233 ms; right before 1673 ms; left after 1325 ms; right after 946 ms; N−: left before 667 ms; right before 838 ms; left after 856 ms; right after 645 ms), as confirmed by the relatively high *η* ^2^ in both groups.

### 3.3. Accuracy

As done for RTs, correct response rates from 13 patients were analysed with a first ANOVA with Time (before versus after the reference) and Distance (1 versus 2 versus 3) as within-subject factors, plus Group (N+ versus N−) as between-subjects factor and mean accuracy as dependent variable. Data from neglect patient N2 were entered (for both mappings) only in this but not in the following ANOVA, as previously done for RTs.

The main effect of Distance was significant, *F*(2,22) = 3.89, *p* < 0.05, and *η* ^2^ = .26, as well as the Time × Distance interaction: *F*(2,22) = 6.89, *p* < 0.01, and *η* ^2^ = .39. Accuracy was similar, for both items before and after the reference, for *d*1 (84%) and *d*2 (90%). In contrast, at *d*3, accuracy was higher for the last (93%) than for the first (82%) item (*t*(12) = 2.13, *p* < 0.05, one-tailed). No other main effects or interactions emerged. A tendency for lower accuracy in N+ (80%) than in N− (94%) was present: *F*(1,11) = 4.57, *p* = 0.056, and *η* ^2^ = .29. In N+ group, the lowest accuracy rates occurred for items 1, 3, and 4 (73%, 75%, and 77%, resp.).

When performing a second ANOVA considering response side (therefore excluding patient N+ 2) the interactions Side × Time, *F*(1,10) = 6.83, *p* < 0.05, and *η* ^2^ = .406, and Time × Distance, *F*(1,10) = 5.46, *p* < 0.05, and *η* ^2^ = .35, were significant. The first interaction was suggestive of a SNARC-like effect, with less errors for left-sided responses to items before the reference (93%) than after the reference (86%), while the opposite effects emerged for right-sided responses (85% accuracy for items before the reference and 95% of accuracy for items after the reference).

The second (Time × Distance) interaction was seemingly due to a difference in accuracy between the two *d*3 values, that is, for the first (88%) and the last item (94%), whereas across distances 1 and 2 the performance was rather similar.

The Side × Time × Group interaction just missed significance: *F*(1, 10) = 3.68, *p* = 0.084, and *η* ^2^ = .27. In N+, accuracy was strongly dependent on the mapping [mean accuracy “incompatible” left-after mapping = 78% (71% if including N+ 2); mean accuracy “compatible” left-before mapping = 93% (90% if including N+ 2)]. This finding mirrors previous studies of neglect within the numerical domain [35, 36] whereby the left-large versus right-small mapping, which is “incompatible” with a left-to-right ordering, leads to a number of errors remarkably higher than the opposite mapping, up to the level of being “impossible” to perform (as occurred for cases 2 and 3 here). In N− patients, this difference was much reduced (mean accuracy left-after mapping = 93%; mean accuracy left-before mapping 96%).

Thus, the analyses on accuracy generally resembled those on RTs, allowing us to rule out that the effects found for RTs were due to a speed-accuracy tradeoff.

## 4. Discussion

We tested a group of right-hemisphere damaged patients, with and without left hemispatial neglect, to study whether the processing of time-ordered events is spatial in nature. Patients were presented with an image depicting one event taken from a story they had memorized. They were asked to categorize this image as occurring before or after an event occurring in the middle of the story. An asymmetric distance effect emerged, in neglect patients only, with slower responses to the images/events that took place before the temporal reference. The first event of the sequence as well as the third one (occurring immediately before the reference) elicited significantly slower responses than their counterparts occurring after the reference. Patients without neglect showed, in contrast, a standard yet symmetric distance effect with respect to the temporal reference.

These findings demonstrate that the representation of events order is spatial in nature and provide strong evidence that ordinality is similarly processed in temporal and numerical sequences. They also confirm that, in left-to-right readers and in the absence of specific manipulations, the left-to-right direction is the preferred axis to spontaneously represent ordinal events in space. Whether the major determinants of this preference mainly are hemispheric asymmetries [47] or linguistic metaphors [48] or reading and writing habits [2] remains to be directly investigated altogether with the role of individual strategies/variability in responding to ordered items.

Within the numerical domain, studies on RHD patients with left neglect have provided crucial evidence supporting a key role for spatial attention in accessing numerical magnitude upon the MNL [36, 49]. Several authors ([37, 50]; see also [51]) have highlighted WM and its interplay with spatial attention as a major determinant of these effects. The effect of a brain damage on the already complex interplay between spatial attention and spatial representations unequivocally leads to a number of dissociations [52]. Strong support for the presence of a spatial representation for numbers comes from the numerical bisection task. When asked to verbally bisect a numerical interval (e.g., what number is halfway between “2” and “6”), patients with left neglect systematically misplace the midpoint of the numerical interval (e.g., responding with “5” instead of “4”). These errors closely resemble the typical pattern found in the bisection of visual lines: that is, patients show increased rightward shifts with increasing line length and a reverse leftward bias (crossover effect) with very short lines [53]. This bias is not directly related to neglect severity in peripersonal space [45, 50, 54] nor in the O'Clock Test [51]. This peculiar form of neglect, which seems to be neither visual nor representational, can be seen as a strong indication that numbers are represented in a way that is spatial in nature [53].

In the numerical cognition domain, the role of ordinality is seldom addressed (for discussion see [52]) and there are only few studies addressing ordinality as a common characteristic belonging to both temporal sequences and numerical quantities. Order processing in fish strikingly suggests that this ability is innate and does not rely on language [55]. The spatial effects characterizing number and time might have a common origin in a spatial representation of order (see [56]). It is, however, also known that order and numerical magnitude processing can dissociate. Turconi et al. [57] described a Gerstmann syndrome patient [58] who performed well in a number comparison task (which number is larger?) but was markedly impaired in a numerical order task (which number comes first?).

With respect to our results, which mechanisms gave rise, in neglect patients, to specific impairments for the items occurring before the reference? Broadly speaking, temporal order responses were selectively slowed in neglect patients and for items occurring “before” the reference. It is important to note that the bias for “what happened before” occurred with significantly asymmetric slowing which emerged selectively for distances 1 and 3 (the first event in the sequence and the one immediately before the reference). Why were positions 1 and 3 disproportionally affected? An explanation might be related to the way the task was solved. It is possible that different representations were generated according to the ordinal position of the presented item. We speculate that when the first item had to be responded to, the whole sequence before the reference was also activated, in such a way that the item was spatially coded as being “left” relative to the whole sequence. On the other hand, when the item immediately before the reference was presented, it might have been spatially coded relative to the reference only. The second item in the sequence would not be double coded as “left” and would then be responded to more quickly. Another alternative explanation might be that item 1 was located to the “leftmost” position in past time and therefore most severely neglected. One might then question why the same slowing for the first item has not been described in the numerical domain for number 1 when the reference is 5 [35–37]. The answer may be that in the numerical domain the presentation of number 1 would not trigger an automatic activation of the numerical range because in that case number 1 only codes for a magnitude and not a “first of many” order. It might then be claimed that when the numerical range is not “canonical,” the first item should lead to slower processing in left neglect because it indexes a “first of many” in the order representation. Surprisingly enough this last prediction is confirmed by previous data [35, Figure 1]; when the reference number was 7 rather than 5, apparently selective slowing for the digits 1 and 2 (versus 3) emerged. Regardless of its origin, the presence of selective slowing for the first item allows ruling out the idea that a spatially characterized slowing occurs only in interaction with the most difficult condition (e.g., distance 1). A final alternative explanation, although more complex, would be that this result reflects an abnormal primacy effect on top of the representational effect for the item immediately preceding the reference. This explanation would however not provide a reason as to why neglect should affect the primacy effect. On the contrary, in order to emerge, the slowing for the first item had to be strong enough to overcome primacy effect. Moreover, it suggests that no verbal labelling of items was implemented to remember the sequence. If not, an advantage rather than a disadvantage for first item(s) should have emerged.

Within the numerical domain, some authors [45] also maintained that the distortions due to neglect in numerical processing reflect impaired access rather than a distorted representation itself. Supporting evidence can be found in [36], whereby spationumerical deficits shown by neglect patients varied according to the task at hand (present in magnitude comparison but absent in parity judgement). Leaving terminological issues aside, it seems that these spatial effects are due to the active “handling” of stimuli. Whether these operations are related to spatial attention shifts and/or to WM processes (see also [7, 59]) will be object of future investigations, altogether with the role of different strategies implemented at individual level.

Our results suggest the presence of a particular representational domain, encompassing both spatial and temporal characteristics, which is affected by neglect. For the moment, it seems unclear whether there is any strict overlap between the spatiotemporal distortions and patients' performance in the physical space. This could not be tested due to the small sample size and variability of neglect tests. Also its relation with the clinical presence/degree of neglect seems puzzling. On the one hand, one could claim that if time is truly spatially represented, a strong quantitative correlation should be expected between neglect severity and time processing. On the other hand, it should be noted that, despite the strong analogies we found, space and time are distinct domains and that it might not be adequate to a priori expect a correlation in performance between the two. Moreover, performance of neglect patients at clinical tests is often the result of the implementation of compensatory strategies, whereas this seems to be less often the case in computer-based tests [26]. Larger-sample studies might shed further light on this issue. The potential absence of a clear correlation between neglect severity and magnitude of the slowing for items “before the reference” may however also suggest another close analogy with the numerical cognition domain, where there is little or no evidence of correlations between neglect severity and behavioural effects in numerical comparison and numerical interval bisection tasks [36, 50].

Also the absence of these spatiotemporal distortions from the standard clinical experience might be explained in analogy with the numerical cognition domain: neglect has little or no impact on “general” numerical abilities. When numerical magnitude has to be processed/ranked (magnitude comparison), dramatic impairments can emerge [36, 53] as opposed to when numerical magnitude is task-irrelevant (party judgment [36]; see also [60]). While the current sample is too small to suggest any reliable lesion to behaviour correlation, it seems worth noting that in most of the patients the parietal lobe was spared.

## 5. Conclusions

Performance of left neglect patients showed selective slowing for items appearing before a time-related reference. While this core finding demonstrates that serial order is spatially coded, four additional conclusions can be also drawn. The first is that while patients show spectacular spatiotemporal distortions, the processing of time as space does not involve neglect patients only. Indeed, healthy participants spontaneously associate early items of a sequence with a left-sided response and late items with a right-sided one [5–7]. In contrast, when the to-be-measured effect involves a shift of attention and single letters are used as sequential stimuli, the results are less clear-cut and might depend on the meaning letters convey. For instance, letters are not associated with a strong ordinal meaning related to their position in the alphabet [61], although letters can interact with the appearance of a lateralized target when they convey order/position in a short-term sequence [62].

A second aspect is the presence, in patients as well as in healthy participants, of overlapping representations for both perceived and conceptual aspects of time. Despite the presumable difference in the cognitive processes involved, very similar effects (for instance, the association of short-before with left and of long-after with right) has been found with experienced as well as with conceptual time [63], again supporting a more general role of spatial attentional operations. At the same time, however, here we describe for the first time that neglect differently affects the processing of the items before a temporal/ordinal reference depending on the ordinal position these items occupy.

A third aspect is related to the demission of the polarity correspondence argument [8] as a viable explanation for order/space associations. Yet, as originally commented by Umiltà [64], the polarity coding can explain potential space-side arbitrary associations when the labelling is verbal. The strikingly different performance showed by neglect patients cannot be attributed to differences in verbal processes.

The last issue concerns the potential broader implications of our findings. By showing that order processing is affected by neglect, our study suggests that neurologically caused spatial deficits present in this syndrome might have widespread consequences, disrupting a number of cognitive aspects well beyond the domains of spatial attention and visual perception. Indeed, given the current evidence that ordinal processing is hampered in neglect, it is not implausible that this deficit might also influence other functions relying on sequential information such as the retrieval of episodic memory. Indeed, the knowledge of order is crucial in many domains and only partly implemented through verbal coding. Based on functional neuroimaging data, it has been proposed that the parietal lobes play a pivotal role in episodic memory [65]. It is tempting to speculate that a mechanism subtending parietal processes in memory would be implemented by spatial attention and shifts between sequential time points. Whereas some evidence of an interplay between spatial attention and episodic memory is already available [66], the two domains might be more closely intertwined than one might think. It might not be inappropriate to think that the loci method for memorizing arbitrary lists of items (cfr. memory palace or mind palace) might build upon a spontaneous tendency of representing order as space.

## Figures and Tables

**Table 1:** 

Group case	Age	Sex	Aetiology	Lesion vol	Days from onset	Bisection [28] % deviation	Bisect. [29] 5 cm	Bisect. [29] 20 cm	Total Om. [30]	L	C	R	Copy [31]	MoCa [32]	Delayed recall (/5)
N+ 1	60	M	H	271885	125	25.04	−0.5	21	22	15	4	3	3	23	4
N+ 2	50	M	H	461428	140	83.72	3	27	28	15	5	8	2	26	5
N+ 3	65	M	H	310108	23	57.20	−2	12	20	14	4	2	3	24	5
N+ 4	70	M	I	455633	45	32.45	9	30	9	9	0	0	1	28	4
N+ 5	52	M	H	15379	156	3 (max 16)^*∗*^	na	na	75^b^	53^b^	nc	22^b^	na	24^MM^	na
N+ 6	59	F	I	45313	60	10 (max 16)^*∗*^	na	na	66^b^	49^b^	nc	17^b^	na	27^MM^	na
N+ 7	72	M	I	56439	23	28.47	3	31	12	8	4	0	1	28	4
N+ 8	60	M	I	116513	65	57.20	8	54	20	14	4	2	1	28	5
N− 1	57	M	H	35377	41	8.65	1	2	0	0	0	0	0	30	5
N− 2	79	M	I	11755	12	7.10	0	3	0	0	0	0	0	27	4
N− 3	32	M	I	18379	18	3.40	2	4	2	1	1	0	0	30	5
N− 4	43	M	I	232185	74	7.10	1	6	4	1	2	1	0	29	4
N− 5	67	M	H	24366	130	4.20	2	1	1	0	0	1	0	29	5
N− 6	67	F	I	245984	34	−0.4	0.8	1	2	2	0	0	0	30	5

*Line bisection 1* [28]: mean error in percentage of maximal possible error. A positive value indicates a rightward error.

*Line bisection 2* [29]: mean left (−) or right (+) deviation in mm for 2 lines of 5 and 20 cm.

*Omissions at bell cancellation task* [30]: number of total omitted bells then separately reported for the left (L; /15), central (C; /5), and right (R; /15) parts of the test sheet.

MM: *MMSE* [33].

*Delayed recall* (MoCA): number of correctly recalled items.

na: not administered.

b: mean of all cancellation tasks from the BIT [34].

nc: not calculated.

^*∗*^BIT [34] aggregated score (sum of bisection, copy, and drawing subtests).
